# FGFR2 and NOTCH1 Expression Inversely Correlated in Progressive Cutaneous Carcinogenesis in an Experimental Mouse Model

**DOI:** 10.3390/jpm14070729

**Published:** 2024-07-05

**Authors:** Georgia Vairaktari, Alexander Schramm, Efstathia Vairaktari, Spyridoula Derka, Andreas Sakkas, Nikolaos Lefantzis, Stavroula Diamantopoulou, Antonis Vylliotis, Andreas Lazaris, Marcel Ebeling, Stavros Vassiliou

**Affiliations:** 1 Department of Oral and Maxillofacial Surgery, University General Hospital Attikon, School of Medicine, National and Kapodistrian University of Athens, 11527 Athens, Greece; 2 Department of Oral and Maxillofacial Surgery, University Hospital Ulm, Albert-Einstein-Allee 10, 89081 Ulm, Germany; 3 Department of Oral and Plastic Maxillofacial Surgery, Military Hospital Ulm, Academic Hospital of the University of Ulm, Oberer Eselsberg 40, 89081 Ulm, Germany; 4 Department of Oral and Maxillofacial Surgery, Evaggelismos General Hospital of Athens, National and Kapodistrian University of Athens, 11527 Athens, Greece; 5 Diagnostic and Research Laboratory of Molecular Biology, BiocLab, 11527 Athens, Greece; 6 Department of Pathology, School of Medicine, National and Kapodistrian University of Athens, 11527 Athens, Greece; alazaris@med.uoa.gr

**Keywords:** skin cancer, experimental carcinogenesis, FGFR2, NOTCH1, immunohistochemistry

## Abstract

Cutaneous squamous cell carcinoma (cSCC) is a common and increasingly prevalent form of skin cancer, posing significant health challenges. Understanding the molecular mechanisms involved in cSCC progression is crucial for developing effective treatments. The primary aim of this research was to evaluate the activation of NOTCH1 and FGFR2 oncogenes in inducing skin cancer in FVB/N mice through a stepwise chemical process. Forty female FVB/N mice, aged four weeks, were randomly divided into a control group (n = 8) and two experimental groups (group A: n = 16, group B: n = 16). This study involved subjecting the groups to a two-stage carcinogenesis procedure. This included an initial application of 97.4 nmol DMBA on shaved skin on their backs, followed by applications of 32.4 nmol TPA after thirteen weeks for group A and after twenty weeks for group B. The control group did not receive any treatment. Their skin conditions were monitored weekly to detect tumor development. After the experiment, the animals were euthanized for further tissue sampling. The examination of skin lesions in the experimental groups showed a correlation with tumor progression, ranging from dysplasia to carcinoma. Tumor samples were assessed both histologically and immunohistochemically. Notably, FGFR2 expression was higher in benign, precancerous, and malignant tumors compared to normal tissue. NOTCH1 expression was only elevated in benign tumors compared to normal tissue. This study demonstrates a clear correlation of FGFR2 expression and the progression of cutaneous neoplasms, while NOTCH 1 expression is inversely correlated in FVB/N mice. This suggests an early involvement of these oncogenes in the development of skin tumors.

## 1. Introduction

Skin cancer is one of the most common types of cancer worldwide, with an increasing incidence over recent decades. It poses significant public health challenges due to its high prevalence and potential for severe outcomes [1,2]. According to recent statistics, millions of new cases are diagnosed annually, highlighting the urgent need for effective prevention and treatment strategies [3].

Among the various forms of skin cancer, cutaneous squamous cell carcinoma (cSCC) is particularly noteworthy for its aggressive nature and potential to metastasize. cSCC arises from the keratinocytes of the epidermis and is influenced by factors such as ultraviolet radiation exposure, immunosuppression, and genetic predispositions [4]. Understanding the molecular mechanisms driving cSCC progression is crucial for developing targeted therapies [5,6].

Recent studies have highlighted the roles of various oncogenes in skin cancer, including NOTCH1 and FGFR2. While NOTCH1 has been extensively studied, the role of FGFR2 in cSCC remains less understood [7,8]. This study aims to evaluate the activation of NOTCH1 and FGFR2 oncogenes in inducing skin cancer in FVB/N mice through a stepwise chemical process, providing new insights into their involvement in cSCC development.

Notch proteins are transmembrane receptors that respond to transmembrane ligands and play a crucial role in various developmental and cellular processes, including carcinogenesis. Upon ligand engagement, Notch undergoes a bipartite proteolytic cleavage cascade, resulting in the release of the Notch Intracellular Domain (NICD) for nuclear translocation. NICD acts as a transcriptional modulator within the nucleus, regulating gene expression [9]. Notch1 is a crucial component of the Notch signaling pathway and has been linked to various types of cancer, including T-acute lymphoblastic leukemia. When hyperactivated or hyperstabilized, Notch1 acts as a proto-oncogene, promoting the initiation and progression of carcinogenesis [10,11]. However, in skin keratinocytes, Notch1 has been attributed a paradoxical ‘tumor suppressor’ role [12,13], although the precise mechanism underlying this unique function remains controversial.

The removal of Notch from the epidermal layer has been linked to the development of cutaneous neoplasms [13,14]. This has led to the identification of Notch1 as a potential tumor suppressor in epidermal biology [13]. However, the exact mechanisms that govern the tumor suppressive role of Notch1 in this context are not yet fully understood. It is also unclear whether other Notch paralogues, such as Notch2 and 3, compensate for its absence in the skin. Notch1’s tumor suppressor activity is proposed to stem from its unique ability to counteract keratinocyte proliferation through one or more cell-autonomous signaling mechanisms. While these insights primarily originate from studies on the precancerous hyperplastic epidermis of Notch-deficient animals [13,14], early changes following Notch loss have not been comprehensively explored.

In vitro studies with isolated keratinocytes were used to draw conclusions [13,15,16]. However, these studies overlooked the complexities of the skin microenvironment and the contributions of other skin components to carcinogenesis.

In vivo studies have shown that the inhibition of Notch signaling during embryonic development leads to epidermal hypoplasia and reduced proliferative capacity in keratinocytes. This suggests that reactive or secondary hyperplasia may underlie the subsequently observed late-stage epidermal hyperproliferation in adult skin lacking Notch activity [17,18]. The molecular alterations described so far may indicate secondary cascades that occur after epidermal hyperplasia in mice that do not have functional Notch signaling.

The Fibroblast Growth Factor Receptor (FGFR) family is a subset of human Receptor Tyrosine Kinases (RTKs), consisting of four extensively conserved transmembrane receptors (FGFR1-4) and one FGF receptor characterized by the absence of an intracellular domain (FGFR5). Although FGFRs are mainly located at the cellular periphery, they can also be found inside organelles such as the nucleus and mitochondria [19]. Cell membrane-bound FGFRs typically have a ligand-binding extracellular domain with three immunoglobulin-like (Ig-like) domains, a transmembrane helical segment, and a cytoplasmic domain containing a catalytically active tyrosine kinase module. Alternative splicing events, particularly in the third Ig-like domain, create a range of FGFR isoforms with different ligand affinities and specificities [20,21].

Fibroblast Growth Factors (FGFs) and their cognate receptors coordinate various physiological processes, including cellular proliferation, differentiation, and motility [22]. Upon phosphorylation of FGFR, several intracellular signaling cascades are activated, including the RAS–RAF–mitogen-activated protein kinase (MAPK)–extracellular regulated kinase (ERK), PI3K/AKT, Stat3, and NFKB pathways, all of which have been linked to skin carcinogenesis [23]. Elevated levels of FGFR have been found in various types of cancer, including prostate, breast, and lung cancers. However, its role in skin cancer remains relatively unexplored [24,25,26]. Previous studies have highlighted the importance of FGFR2 gene amplification, aberrant activation, or single-nucleotide polymorphisms in driving oncogenic progression [27,28,29]. In our previous research involving SKH-1 mice, exposure to UVB radiation resulted in increased FGFR2 phosphorylation in the skin. Preemptive treatment with AZD4547, a selective FGFR inhibitor, significantly reduced UVB-induced FGFR2 activation, thereby reducing UVB-triggered epidermal hyperplasia and hyperproliferation, which are key early events in UVB-induced carcinogenesis [30]. However, the potential therapeutic use of FGFRs in modulating the promotion and progression of cutaneous squamous cell carcinoma (cSCC) has yet to be explored.

Several studies have shown that UVB radiation can activate the mTOR pathway in skin keratinocytes [31,32,33,34,35]. The mTOR pathway is a crucial serine/threonine kinase that plays various roles, including cellular stress responses, growth factor signaling, nutrient sensing, and stress signal transduction [36]. Photocarcinogenesis involves the inhibition of apoptosis and the stimulation of proliferation through mTOR, which is often enhanced by upstream signals such as Ras or phosphoinositide 3-kinase (PI3K). This leads to increased proliferation and reduced susceptibility to apoptotic stimuli [37]. The mTOR pathway consists of rapamycin-sensitive mTORC1 and rapamycin-resistant mTORC2 complexes. In epidermal tumors such as cutaneous squamous cell carcinoma (cSCC) and precancerous actinic keratoses (AKs), the mTOR pathway exhibits elevated phosphorylation levels of mTOR, AKT, and their downstream effectors in comparison to normal skin [38]. UVB-induced phosphorylation events targeting 4EBP1, S6K, and AKT underscore the pivotal role of the mTOR pathway in fostering tumorigenesis. FGFR2 transmits regulatory signals to downstream effectors by modulating the mTOR pathway. In mouse embryonic fibroblasts, mTOR activation suppresses autophagy facilitated by FGF receptor substrate 2α (FRS2α) and PI3K/AKT, highlighting the essential role of mTOR activation in mediating FGF’s autophagy-suppressive effects [39].

The primary objective of this investigation was to assess the expression patterns of the NOTCH1 and FGFR2 genes and to explore potential correlations between them across successive histological stages of cutaneous squamous cell carcinoma development in an experimental animal model of skin carcinogenesis. A secondary objective was to assess the utility of our two-stage carcinogenesis protocol in elucidating the involvement of additional genes in skin oncogenesis. Notably, this study represents the first and novel investigation of these objectives in a mouse model.

## 2. Materials and Methods

### 2.1. Animals

Forty female FVB/N mice, aged four weeks and weighing approximately 100 g each, were obtained from the Hellenic Pasteur Institute in Athens, Greece. The FVB strain is an inbred laboratory mouse lineage named for its susceptibility to Friend leukemia virus B. It is highly favored in transgenic research due to its large litter sizes and oocytes with prominent pronuclei. Notably, this distinctive phenotype is only present in oocytes and not in sperm. The FVB/N strain was chosen for its susceptibility to skin tumors, as documented in prior studies, making it suitable for the current investigation. Age-matched female mice were selected due to the proclivity for male aggression and the impracticality of group housing male cohorts [40].

The mice were handled in accordance with the ethical standards outlined in the Guide for the Care and Use of Laboratory Animals: Eighth Edition [41] After a two-week acclimatization period in our facility, during which they were provided with rodent chow and ad libitum access to tap water, the animals were randomly assigned to four groups: a control group (group C: n = 8) and two experimental groups for carcinogen treatment (group A: n = 16, group B: n = 16). Rigorous randomization procedures were implemented to ensure unbiased group allocation, and the study design meticulously adhered to the ARRIVE guidelines, emphasizing transparency and reproducibility in research.

For optimal housing conditions, four mice were placed in each cage, which was lined with corn cob bedding and subjected to a 12 h light/dark cycle. Each animal was uniquely identified through an ear tattoo code, ensuring traceability throughout the study period. This approach to experimental design, housing, and identification is crucial in maintaining the welfare of the animals and upholding the scientific rigor of the investigation.

### 2.2. Two-Stage Carcinogenesis Protocol

Forty female FVB/N mice, aged four weeks, underwent dorsal surface shaving using electric clippers to determine their hair cycle phase. After assessment, all mice were found to be in the telogen phase and were included in the study according to the criteria established by Mardaryev [40].

One week after hair removal, the animals in groups A (n = 16) and B (n = 16) were anesthetized with ether and topically treated on the dorsal skin with 97.4 nmol of the initiator 7,12-dimethylbenz[a]anthracene (DMBA) using a #4 camel’s hairbrush (Sigma, St. Louis, MO, USA). A repeated application regimen involving 32.4 nmol of the tumor promoter 12-O-tetradecanoyl phorbol-13-acetate (TPA) was administered twice within a four-day interval per week [42]. The TPA treatment duration spanned 13 weeks for group A and 20 weeks for group B, aligning with the regimen proposed by Abel et al. [43]. The control group, denoted as group C (n = 8), received no applications.

**Figure 1:**
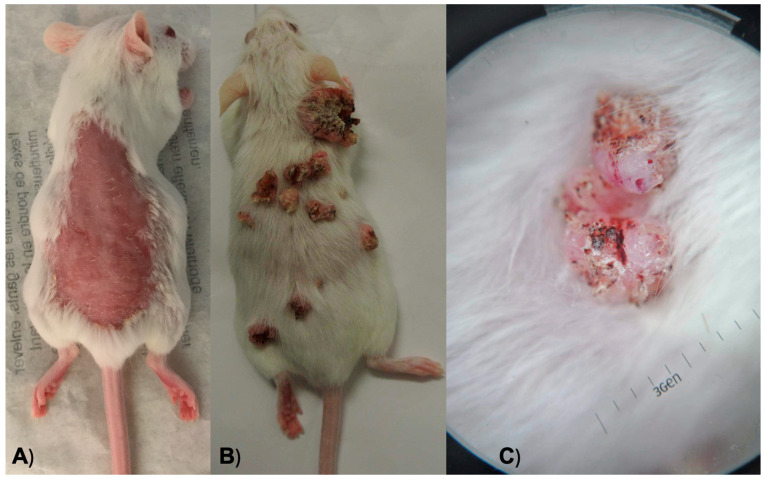
(**A**) An anesthetized mouse after hair removal from its back. (**B**) A mouse during a weekly examination to observe its lesions and tumor growth. (**C**) Dermoscopy of one of the neoplasms on a mouse’s skin.

**Figure 2:**
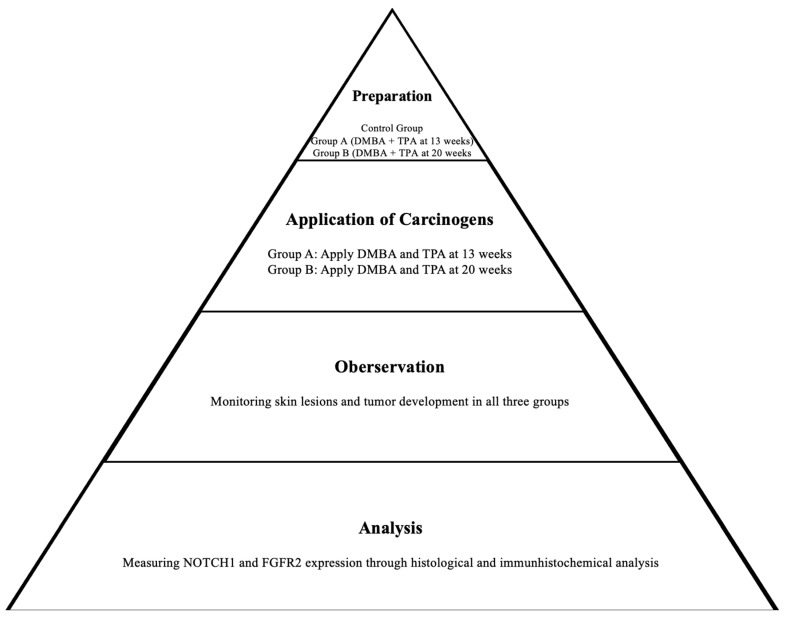
Scheme of the two-stage carcinogenesis protocol.

Weekly examinations were conducted to observe the lesions and tumor growth on the animal skin. Dermatological assessments were performed by two experienced dermatologists in the field of skin cancer, following the methodology outlined by Quintanilla et al. [44], which included observation, palpation, and dermatoscopy. Any palpable mass exceeding 1 mm in size that persisted for more than 2 weeks was meticulously recorded, following the criteria established by DiGiovanni [45] (Figure 1 and Figure 2).

Finally, all animals were euthanized via isoflurane overdose. Visible lesions on the treated skin were excised either fourteen weeks (group A) or twenty-one weeks (group B) after the application of the carcinogen. The tumor sizes were approximately 1 cm in most cases. Furthermore, at fourteen weeks, the control group C was euthanized, and a tissue sample was excised from their dorsal skin. All samples collected from groups A, B, and C were assigned numbers and evaluated impartially.

### 2.3. Histopathological Analysis

A cohort of 459 biopsy samples were fixed in a 10% neutralized formaldehyde solution and embedded in paraffin. Three 4 mm sections were then prepared from each specimen and affixed to Super Frost Plus-coated glass slides (Menzel and Co., Braunschweig, Germany). For the standard histological analysis, one section was stained with hematoxylin and eosin, while the other two sections were used for the immunohistochemical detection of NOTCH1 and FGFR2 gene products.

The histological examination involved a thorough analysis of the entire section under light microscopy, enabling the classification of tissue profiles into distinct categories. The categories assessed in this study included normal, hyperkeratosis, hyperplasia, dysplasia (low grade and high grade), papillomas, in situ carcinoma, well-differentiated squamous cell carcinoma, and poorly differentiated squamous cell carcinoma (refer to Table 1 for additional information). It is important to note that each sample was meticulously evaluated, with all distinct lesions assessed and categorized independently. This rigorous histopathological approach ensures a detailed and nuanced understanding of the varied tissue alterations present in the biopsy samples. For benign tumors, we defined criteria such as well-demarcated borders, the absence of invasion into surrounding tissues, and resemblance to a normal tissue architecture under microscopic examination. Malignant tumors were classified based on criteria including cellular pleomorphism, invasion into adjacent tissues, and the presence of mitotic figures. Precancerous lesions were identified by cellular atypia, dysplastic changes, and early signs of abnormal growth patterns without invasion. These criteria were applied consistently across all samples by a trained histopathologist to ensure robust classification.

### 2.4. Immunohistochemical Analysis

The tissue sections were incubated with antibodies targeting NOTCH1 and FGFR2. The FGFR2 and NOTCH1 polyclonal antibodies (PA1-31990 and MA1-91405) are recommended for detecting FGFR2 and NOTCH1 of human and mouse origin using various techniques, including Western blot (WB), immunohistochemistry (Paraffin) (IHC (P)), immunocytochemistry (ICC/IF), and flow cytometry (Flow), at concentrations of 0.5 and 0.2 mg/mL, respectively. The antibodies used in this study were sourced from Thermo Fisher Scientific.

The incubation process followed the standard immunohistochemical methodology, as outlined by Derka et al. (2006). Positive controls for NOTCH1 and FGFR2 included a pancreas with robust NOTCH1 expression and a liver with strong FGFR2 expression. These tissues were selected based on their well-established expression profiles of NOTCH1 and FGFR2, ensuring that our antibodies were correctly detecting the target proteins in our experimental conditions. In parallel, negative controls were processed alongside the experimental samples, where phosphate-buffered saline (PBS) was substituted for the primary antibody. This step was crucial in confirming the specificity of our immunohistochemical staining, ensuring that any observed staining was attributable solely to the presence of NOTCH1 and FGFR2 proteins and not to non-specific interactions or background staining.

To ensure an unbiased evaluation, two investigators who were blinded to the experimental conditions independently reviewed all samples. This meticulous approach to immunohistochemical analysis, which incorporated positive and negative controls, reinforces the reliability and validity of the study’s findings.

### 2.5. Statistical Analysis

Positive percentage values were calculated for each distinct lesion within each sample. These values were then organized into tables for each animal group (control group and experimental groups A and B) and compared. To analyze the distribution pattern of antibody expression in relation to histological status, the lesions were categorized as histologically normal, precancerous lesions, benign tumors, or malignant tumors. To contrast the percentages of EGFR- and HER2-positive expression between groups A and B against the control group, as well as between histologically normal specimens and those exhibiting precancerous, benign, and malignant characteristics, the non-parametric Mann–Whitney test was employed due to the violation of normality assumptions as per the Kolmogorov–Smirnov criterion. All reported *p*-values were derived from two-tailed tests. Statistical significance was determined at a significance level of *p* < 0.05. The analyses were performed using SPSS software version 26.0.

## 3. Results

The data from 459 biopsies were analyzed (8 from the control group, 211 from group A, and 240 from group B). Their histological statuses are presented in Table 1 separately. 

**Table 1:** Histological status of biopsies in the control and the two experimental groups.

	Group					
	Control		A		B	
	Ν	%	Ν	%	Ν	%
Normal histology	8	100.0	6	2.8	2	0.8
Malignant tumors	0	0.0	9	4.3	8	3.3
Precancerous lesions	0	0.0	162	76.8	151	62.9
Benign tumors	0	0.0	34	16.1	79	32.9

The percentage of NOTCH1-positive expression per animal is presented in Table 2, by group. 

**Figure 3:**
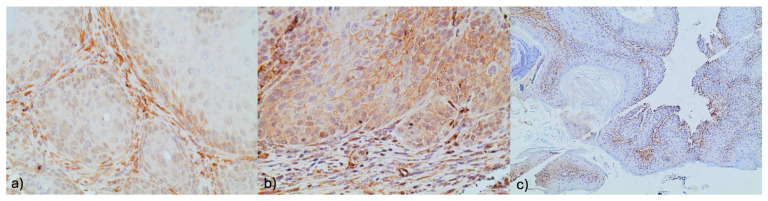
(**a**) NOTCH 1 stromal immunoreactivity with concomitant membranous NOTCH 1 immunoreactivity in an adjacent focus of dysplasia (×400). (**b**) NOTCH1 focal stromal immunoreactivity (×100). (**c**) Membranous Notch1 immunoreactivity in squamous cells (×400).

The mean percentage of positive expression for the control group was 0% (SD = 0%); for group A, it was 34.1% (SD = 25.1%); and for group B, it was 53.2% (SD = 17.8%) (Figure 3). 

The mean percentages of NOTCH1-positive expression in groups A and B were significantly higher compared to the control group (*p* < 0.001 for both comparisons). In group B, the mean percentage was significantly higher compared to group A (*p* = 0.010).

The percentage of FGFR2-positive expression per animal is presented in Table 3, for each group separately. 

**Figure 4:**
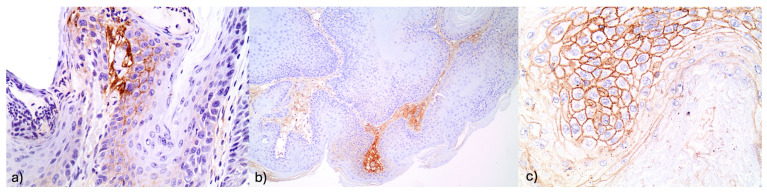
(**a**) Increased FGFR2 expression in stromal elements (×400). (**b**) Intense FGFR2 expression in stromal and adjacent squamous cells (×400). (**c**) Increased FGFR@ stromal immunoexpression in a hyperplastic lesion (×100).

The mean percentage of positive expression for the control group was 0% (SD = 0%); for group A, it was 41.5% (SD = 24.6%); and for group B, it was 58.8% (SD = 25.7%) (Figure 4). 

The mean percentages of FGFR2-positive expression in groups A and B were significantly higher compared to the control group (*p* < 0.001 for both comparisons), while between groups A and B, the percentage of positive expression tended to be higher in group B (*p* = 0.054).

The percentages of NOTCH1- and FGFR2-positive expression, according to their histological status, are presented in Table 4. 

The mean percentage of NOTCH1-positive expression was 18.8% (standard deviation (SD) = 40.3%) in normal histology, 41.2% (SD = 50.7%) in malignant tumors, 44.6% (SD = 49.8%) in precancerous tumors, and 47.7% (SD = 50.2%) in benign tumors. A comparison of the mean percentages of NOTCH1-positive expression between histological statuses revealed that the positive expression in precancerous and benign tumors was significantly higher than that in normal histology (*p* = 0.043 and *p* = 0.030, respectively). Furthermore, the mean percentage of NOTCH1-positive expression in malignant tumors was comparable to that of normal cases (*p* = 0.168), and precancerous and benign tumors (*p* = 0.0.785 and *p* = 0.615, respectively). The mean percentage of NOTCH1-positive expression in benign tumors was comparable to that in precancerous lesions (*p* = 0.562). In the total sample, the mean percentage of FGFR2-positive cells was significantly greater than the corresponding percentage of NOTCH1-positive cells. This finding was also significant in malignant tumors and precancerous lesions, while no significant differences were found in normal histology and benign tumors.

The mean percentage of FGFR2-positive expression was 12.5% (SD = 34.2%) in normal histology, 76.5% (SD = 43.7%) in malignant tumors, 52.2% (SD = 50%) in precancerous lesions, and 50.4% (SD = 50.2%) in benign tumors. A significantly higher mean percentage of FGFR2-positive expression was found in malignant, precancerous, and benign tumors compared to normal histology (*p* < 0.001, *p* = 0.002, and *p* = 0.008, respectively). Furthermore, the mean percentage of FGFR2-positive expression in malignant tumors was significantly greater than in precancerous and benign tumors (*p* = 0.050 and *p* = 0.046, respectively). The mean percentage of FGFR2-positive expression in benign tumors was found to be similar to that observed in precancerous tumors (*p* = 0.743).

To further support our findings, we conducted a detailed analysis of the expression patterns of NOTCH1 and FGFR2 across different histological stages. The mean percentage of NOTCH1-positive cells was highest in benign tumors (47.7%) and lowest in malignant tumors (18.8%), with precancerous lesions showing intermediate levels (41.2%). Conversely, FGFR2-positive cells were most abundant in precancerous (76.5%) and malignant tumors (52.2%), with lower levels in benign tumors (50.4%). These data underscore the inverse correlation between NOTCH1 and FGFR2 expression as tumors progress from the benign to malignant stages (Table 4).

We have calculated both Spearman and Pearson correlation coefficients for NOTCH1 and FGFR2 expression in relation to tumor progression. The Spearman correlation coefficients are as follows: NOTCH1 and FGFR2 expression, 0.988; NOTCH1 expression and tumor progression, 0.953; and FGFR2 expression and tumor progression, 0.953. The Pearson correlation coefficients are as follows: NOTCH1 and FGFR2 expression, 0.988; NOTCH1 expression and tumor progression, 0.950; and FGFR2 expression and tumor progression, 0.948. These correlation coefficients indicate a strong positive correlation between NOTCH1 and FGFR2 expression and their relationship with tumor progression, supporting our reported findings.

## 4. Discussion

The intent of this study was to evaluate the expression patterns of the NOTCH1 and FGFR2 genes and to explore potential correlations between them across successive histological stages of cutaneous squamous cell carcinoma. It was assumed that the expression of the investigated genes increases as the degree of tissue dedifferentiation increases. This hypothesis was at least partially confirmed. The mouse model for skin carcinogenesis has played a crucial role in understanding how human epithelial cancers develop [45,46]. By conducting multistage experiments on mice skin, researchers can gain insights applicable to both scientific investigation and clinical treatment, potentially leading to innovative approaches in managing skin cancer. The identification of oncogenes in human cancers has significantly advanced our comprehension of the disease, with these molecular markers holding promise for therapy, early detection, and prognosis. Our research consortium has developed animal models to study the molecular alterations leading to the onset of oral squamous cell carcinomas in rodents. These models were pioneered based on our prior expertise [47,48,49,50]. Based on this foundational knowledge, we conducted a two-stage in vivo carcinogenesis investigation using FVB/N mice skin as our experimental substrate. Our primary focus was to determine the expression patterns of NOTCH1 and FGFR2 oncogenes across all discernible histological stages. The examination of skin lesions in our experimental groups closely followed the progression of tumor development, from dysplasia to poorly differentiated carcinoma.

It could be shown that a longer treatment with 7,12-dimethylbenz[a]anthracene (DMBA) and 12-O-tetradecanoyl phorbol-13-acetate (TPA) leads to a percentage increase in the development of malignant lesions. 

In our study, we observed distinct expression patterns of NOTCH1 and FGFR2 across different histological stages of cutaneous squamous cell carcinoma (cSCC). Notably, NOTCH1 expression was higher in benign tumors compared to precancerous and malignant tumors, whereas FGFR2 expression showed an opposite trend, with higher levels in precancerous and malignant tumors compared to benign tumors. The elevated NOTCH1 expression in benign tumors suggests a potential role in the early stages of tumorigenesis. Previous studies have indicated that NOTCH1 can function as a tumor suppressor in skin, where its activation may inhibit keratinocyte proliferation and promote differentiation. This tumor-suppressive activity could explain the higher NOTCH1 levels observed in benign tumors, which are less aggressive and more differentiated compared to its malignant counterparts. For example, Radtke et al. demonstrated that Notch1 deficiency in skin and primary keratinocytes leads to the development of basal-cell carcinoma-like tumors due to sustained Gli2 expression [12]. 

In contrast, FGFR2 was significantly elevated in precancerous and malignant tumors, indicating its role in the progression and aggressiveness of cSCC. FGFR2 activation is known to promote oncogenic pathways such as the MAPK and PI3K/AKT signaling cascades, which enhance cell proliferation, survival, and migration. The high FGFR2 expression in more advanced tumor stages aligns with its role in driving tumorigenesis and supporting the malignant phenotype. Grose et al. found that conditional FGFR2b knockout in murine epidermis increased SCC incidence, underscoring FGFR2’s role in tumor development. The observed opposite trends in NOTCH1 and FGFR2 expression can be attributed to their distinct roles in tumor biology. While NOTCH1’s tumor-suppressive functions are more prominent in the initial, less aggressive stages of tumor formation, FGFR2’s oncogenic activities drive tumor progression and malignancy. This dichotomy highlights the complex interplay between different signaling pathways in cSCC and suggests potential therapeutic targets at various stages of the disease.

Our findings on the involvement of NOTCH1 in skin cancer development are consistent with those in the current literature. 

Wang et al. demonstrated through immunofluorescence and Western blot analysis that NOTCH1 is upregulated in skin cancer tissues [51]. The inhibition or activation of NOTCH1 resulted in an altered expression of E-cadherin in the experimental setup, such as changes in DNA methylation. Moreover, it has been demonstrated that the migration and invasion of tumor cells are affected by the activation or inhibition of NOTCH1. Therefore, NOTCH1 is a promising target for personalized and targeted therapies for skin cancer. Kopan et al. conducted experiments that revealed that the non-cell autonomous consequences of defective barrier formation are responsible for the tumor-promoting effects of Notch1 loss in mouse skin. 

However, the understanding of FGFR2 expression remains unclear. FGFR2 is a receptor tyrosine kinase implicated in various cellular processes, including proliferation, differentiation, and survival. Aberrant FGFR2 signaling has been linked to tumorigenesis in several cancers, including breast, gastric, and endometrial cancers, through the activation of downstream pathways such as MAPK, PI3K/AKT, and STAT signaling cascades. In the context of cSCC, FGFR2 gene amplification and mutations can lead to its constitutive activation, promoting oncogenic pathways that contribute to skin carcinogenesis [52]. Recent studies have shown that selective FGFR inhibitors, such as AZD4547, can reduce UVB-induced FGFR2 activation and subsequent epidermal hyperplasia, highlighting its potential as a therapeutic target in cSCC. Despite these findings, further research is needed to fully understand the specific role and mechanisms of FGFR2 in cSCC [24]. We have updated the Section 4 Discussion to include these insights and relevant references to provide a more comprehensive understanding of FGFR2’s involvement in cSCC. Grose et al. demonstrated that conditional FGFR2b knockout within the epidermis of murine models resulted in an increased incidence of SCCs [52], which contrasts with Nan et al.’s findings. Nan et al.’s investigation found no evidence implicating genetic variants in FGFR2 or FGFR4 genes in contributing to the hereditary predisposition to skin cancer among Caucasian women [53].

While our study provides significant insights into the expression patterns of NOTCH1 and FGFR2 in cutaneous squamous cell carcinoma (cSCC), there are several limitations to consider. The sample size, especially of the control group, was relatively small, which might affect the robustness of the statistical analyses. Future research should aim to include larger sample sizes and more balanced group distributions to ensure more robust and statistically sound comparisons. A further limitation of this study is the exclusive use of female FVB/N mice. This was a deliberate choice to maintain consistency in hormonal profiles and to avoid potential hormonal fluctuations in male mice. However, this restricts the generalizability of our findings to male subjects. Future studies incorporating male mice would be essential to comprehensively understand any potential sex-specific differences in the activation of NOTCH1 and FGFR2 oncogenes and their role in skin cancer induction. Furthermore, investigating the impact of sex hormones on these pathways could offer valuable insights into the underlying mechanisms of oncogene activation in skin carcinogenesis, despite the challenges of housing male mice and the potential for aggression. In conclusion, while our findings are promising, they highlight the need for additional research to explore these limitations. Future studies should focus on incorporating a broader range of samples, including both sexes, and employing molecular techniques to deepen our understanding of the genetic and epigenetic mechanisms driving cSCC.

However, it is important to interpret these findings cautiously due to the limitations discussed above. The harvested data support the hypotheses of activity of Notch1 and FGFR2 throughout various stages of tumorigenesis, spanning from initial oncogenic phases to advanced malignancies. Additionally, a clear correlation between FGFR2 and the progression of cutaneous neoplasms is revealed, while NOTCH 1 is inversely correlated. This may encourage further research. If these results are confirmed in future applications for the diagnosis, treatment, or prognosis of skin cancer, they may become more significant. 

## 5. Conclusions

The animal model showcased here holds promise as a valuable resource for unraveling the involvement of additional genes in skin cancer development. Our study revealed a clear correlation of FGFR2 and the progression of cutaneous neoplasms, while NOTCH 1 is inversely correlated. These results hint at a potential early engagement of these oncogenes in driving the advancement of skin tumors within this model. Nevertheless, further studies with male and female mice are necessary to validate our results in the future.

## Figures and Tables

**Table 2:** Percentage of NOTCH1-positive cells in the control group and in the two experimental groups.

NOTCH1	Control Group	Group A	Group Β
	0.0	5.0	45.5
	0.0	15.4	52.9
	0.0	43.8	60.0
	0.0	6.7	33.3
	0.0	27.3	60.0
	0.0	50.0	57.1
	0.0	50.0	0.0
	0.0	0.0	60.0
		62.5	62.5
		41.2	45.5
		22.2	75.0
		88.9	50.0
		40.0	62.5
		0.0	62.5
		57.1	75.0
		35.3	50.0
Mean percentage (SD)	0.0 (0.0)	34.1 (25.1)	53.2 (17.8)
Median percentage (IQR)	0.0 (0.0–0.0)	37.7 (11.1–50.0)	58.6 (47.8–62.5)
*p* +	-	<0.001	<0.001

+ *p*-value from the Mann–Whitney test for comparison with the control group.

**Table 3:** Percentage of FGFR2-positive cells in the control group and in the two experimental groups.

FGFR2	Control Group	Group A	Group Β
	0.0	57.1	36.4
	0.0	53.8	82.4
	0.0	75.0	100.0
	0.0	26.7	33.3
	0.0	31.8	80.0
	0.0	50.0	54.3
	0.0	62.5	13.3
	0.0	75.0	64.0
		25.0	50.0
		23.5	75.0
		77.8	12.5
		44.4	66.7
		0.0	75.0
		0.0	50.0
		28.6	90.9
		33.3	57.1
Mean percentage (SD)	0.0 (0.0)	41.5 (24.6)	58.8 (25.7)
Median percentage (IQR)	0.0 (0.0–0.0)	38.9 (25.9–59.8)	60.6 (43.2–77.5)
*p* +	-	<0.001	<0.001

+ *p*-value from the Mann–Whitney test for comparison with the control group.

**Table 4:** Percentages of NOTCH1- and FGFR2-positive cells according to histological status.

	Total Sample	Histological Status			
		Normal Histology	Malignant Tumors	Precancerous Lesions	Benign Tumors
	(N = 459)	(N = 16)	(N = 17)	(N = 313)	(N = 113)
NOTCH1					
Mean percentage (SD)	44.3 (49.7)	18.8 (40.3)	41.2 (50.7)	44.6 (49.8)	47.7 (50.2)
Median percentage (IQR)	0 (0–100)	0 (0–0)	0 (0–100)	0 (0–100)	0 (0–100)
*p* +			0.168	0.043	0.030
FGFR2					
Mean percentage (SD)	51.3 (50.0)	12.5 (34.2)	76.5 (43.7)	52.2 (50)	50.4 (50.2)
Median percentage (IQR)	100 (0–100)	0 (0–0)	100 (100–100)	100 (0–100)	100 (0–100)
*p* +			<0.001	0.002	0.005
*p* ++	0.028	0.564	0.014	0.046	0.763

+ *p*-value from the Mann–Whitney test for comparison with normal histological status. ++ *p*-value from the Wilcoxon signed-rank test for comparison between NOTCH1- and FGFR2-positive cells.

## Data Availability

The data are available from the corresponding author on reasonable request.
